# Phase 1 study evaluating safety and pharmacokinetics of tusamitamab ravtansine monotherapy in Japanese patients with advanced malignant solid tumors

**DOI:** 10.1007/s10147-025-02784-4

**Published:** 2025-05-25

**Authors:** Kei Muro, Kentaro Yamazaki, Shigenori Kadowaki, Saori Mishima, Takeshi Kawakami, Tomoyuki Tanaka, Keisuke Tada, Nathalie Fagniez, Shinobu Ohshima, Takayuki Yoshino

**Affiliations:** 1 Department of Clinical Oncology, Aichi Cancer Center Hospital, Nagoya, Japan; 2 Division of Gastrointestinal Oncology, Shizuoka Cancer Center, Shizuoka, Japan; 3 Department of Gastrointestinal Oncology, National Cancer Center Hospital East, Kashiwa, Japan; 4 Sanofi K.K., Tokyo, Japan; 5 Sanofi Pharmacokinetics, Dynamics and Metabolism, Vitry-sur-Seine, France

**Keywords:** Antibody drug conjugates, CEACAM5, Tusamitamab ravtansine, Japanese adults, Advanced solid tumors

## Abstract

**Background:**

Tusamitamab ravtansine (SAR408701) is an immunoconjugate that binds carcinoembryonic antigen-related cell adhesion molecule 5 (CEACAM5) and delivers its cytotoxic payload to target cells. Here, we report findings from three dosing regimens of tusamitamab ravtansine administration in Japanese adults with advanced malignant solid tumors.

**Methods:**

Japanese adults (aged ≥ 20 years) with CEACAM5-expressing malignant solid tumors were enrolled in this Phase 1, open-label, non-randomized, dose-escalation evaluation of tusamitamab ravtansine in three parts: (i) main dose-escalation part with every two weeks (Q2W) administration, (ii) loading dose (LD) part with Q2W administration with a LD at Cycle 1 (C1) only, and (iii) dose-escalation every three weeks (Q3W) part. Primary objectives were to evaluate the tolerability and safety of tusamitamab ravtansine.

**Results:**

Nine patients were enrolled in the main dose-escalation part, 16 patients in the dose-escalation *bis* part with LD, and nine patients in the dose-escalation Q3W part. Administration of tusamitamab ravtansine resulted in a manageable safety profile with no dose-limiting toxicities reported during the observation period except for two events during dose-escalation *bis* Q2W part. Most common adverse events (AEs) were corneal events, gastrointestinal disorders, and metabolic events. After first administration, tusamitamab ravtansine exposure was dose proportional over the dose range 80–170 mg/m^2^. Best overall response (BOR) was stable disease, observed in all three parts; confirmed response was not observed at any dose level.

**Conclusion:**

Tusamitamab ravtansine demonstrated a tolerable safety profile at a dose of 80–170 mg/m^2^ in three different administration schedules in Japanese adults with metastatic solid tumors.

## Introduction

Carcinoembryonic antigen (CEA)-related cell adhesion molecule 5 (CEACAM5, also known as CD66e), a cell surface glycoprotein, is reportedly upregulated in several tumor types, including non-squamous non-small-cell lung cancer, and plays a key role in tumor progression by inhibiting apoptosis, blocking cell differentiation, and regulating metastasis [1, 2]. Elevated expression of CEACAM5 is associated with poor survival in patients with colorectal cancer and gastric cancer [3–5]. Given its differential expression in tumors compared with normal tissues, CEACAM5 presents as an attractive therapeutic target.

Antibody–drug conjugates (ADCs) have recently emerged as one of the key therapeutic modalities in the tumor therapy landscape. ADCs comprise a monoclonal antibody covalently attached to a cytotoxic drug via a chemical linker. Currently approved ADCs target multiple indications, including alternative tumor types, and have demonstrated remarkable potential for replacing conventional chemotherapy [6, 7].

Tusamitamab ravtansine (SAR408701) is an ADC comprising an anti-CEACAM5 antibody that is covalently bound to the maytansinoid derivative 4 (DM4) through an N-succinimidyl 4-(2-pyridyldithio)-butyrate linker that is stable in plasma. Maytansine and its derivatives are potent anti-tumor agents that inhibit tumor cell proliferation by disrupting microtubule assembly during mitosis [8]. Binding of tusamitamab ravtansine to CEACAM5-expressing tumor cells leads to the internalization of the complex into endosomes. Following lysosome trafficking of the endosome, the acidic lysosomal environment cleaves the linker site, resulting in the release of cytotoxic DM4 derivatives into the cytoplasm wherein they bind with the β-subunit of tubulin and initiate microtubule depolymerization and, consequently, cell cycle arrest [8].

Tusamitamab ravtansine has demonstrated anti-tumor activity in CEACAM5-expressing tumor cell lines and patient-derived xenograft mouse models [9]. In a previous first-in-human (FIH) study (NCT02187848), we reported an acceptable safety profile in patients with locally advanced/metastatic solid tumors treated with tusamitamab ravtansine when administered in a fixed-dose regimen (5–150 mg/m^2^ every two weeks [Q2 W]), a loading dose (LD) regimen (120–170 mg/m^2^ followed by 100 mg/m^2^ Q2 W), and in another fixed-dose regimen (120–170 mg/m^2^ every three weeks [Q3 W]); maximum tolerated doses (MTDs) were 100 mg/m^2^ Q2 W, 170 mg/m^2^ (LD) followed by 100 mg/m^2^ Q2 W, and 170 mg/m^2^ Q3 W, respectively [10, 11].

Corneal events (Grade 2 or 3 keratopathy and keratitis) and increased transaminases were the observed dose-limiting toxicities (DLTs) in the FIH study. A Grade 3 increase in transaminase levels during Cycle (C) 1 in the Q3 W cohort prompted the withdrawal of therapy. All DLTs were non-fatal and reversible following dose modification (cycle delays or dose reductions). Asthenia, nausea, abdominal pain, and keratopathy were the common treatment-emergent adverse events (TEAEs) reported in both cohorts. Objective response was observed in three patients in the fixed-dosing regimen Q2 W cohort [10, 11].

The objective of the present Phase 1, dose-escalation study was to evaluate the safety, pharmacokinetics (PK), and anti-tumor activity of the dose regimens used in the FIH study in Japanese adults with advanced solid tumors (NCT03324113) expressing CEACAM5 or with elevated levels of circulating CEA.

## Patients and methods

### Study design

**Fig. 1:**
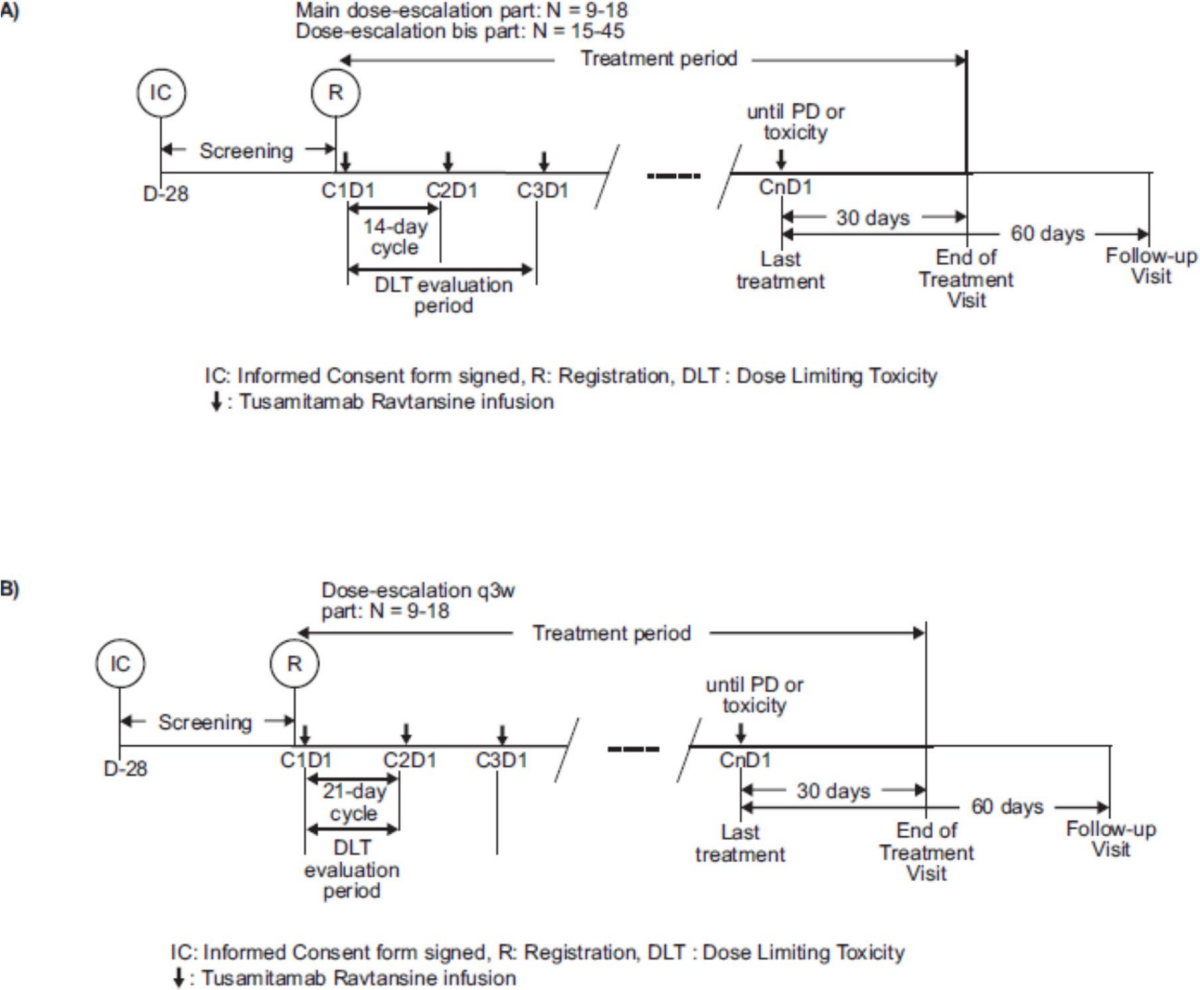
**A** Treatment schema for dose-escalation part (main and *bis* with loading dose); **B** Treatment schema for the dose-escalation Q3 W part. *C* cycle; *D* day; *DLT* dose limiting toxicity; *IC* informed consent; *PD* progressive disease; *Q2 W* every two weeks; *Q3 W* every three weeks; *R* registration

This was a Phase 1, open-label, non-randomized, dose-escalation evaluation of tusamitamab ravtansine administered intravenously as monotherapy (Q2 W or Q3 W) in Japanese adults with advanced solid tumors (NCT03324113). The study comprised three different dose-escalation parts: (i) a main dose-escalation part with Q2 W administration of tusamitamab ravtansine (Fig. 1a); (ii) an LD part with Q2 W administration of tusamitamab ravtansine with a LD at C1, followed by 100 mg/m^2^ Q2 W (Fig. 1a); and (iii) a dose-escalation Q3 W part with Q3 W administration of tusamitamab ravtansine (Fig. 1b). The decision to escalate to the next dose level (DL) was based on the results of the FIH Phase 1 study (NCT02187848), and the dose-escalation decision rule was defined using a modified toxicity probability interval-2 (mTPI-2) approach.

The study was conducted in accordance with the Declaration of Helsinki, International Council for Harmonisation of Technical Requirements for Pharmaceuticals for Human Use, and Good Clinical Practice guidelines. The protocol and all amendments were approved by the Ethics Committee or Institutional Review Board at each study site. All patients provided written informed consent before participating in the trial.

### Eligibility criteria

#### Inclusion criteria

Eligible Japanese patients (aged ≥ 20 years) had CEACAM5-expressing locally advanced or metastatic solid malignant tumors. CEACAM5 expression was assessed retrospectively using immunohistochemistry on the most recent formalin-fixed paraffin-embedded archival tissue samples. Circulating CEACAM5 expression levels were used for enrichment.

#### Exclusion criteria

Patients with an Eastern Cooperative Oncology Group (ECOG) performance status of ≥ 2, a life expectancy of < 12 weeks, and a prior therapy targeting CEACAM5 or prior maytansinoid treatment were excluded from the study. Detailed inclusion and exclusion criteria are mentioned in Supplementary Table 1.

### Study interventions

Tusamitamab ravtansine was administered intravenously on Day (D) 1 and repeated Q2 W for the main dose-escalation and dose-escalation parts with LD or repeated Q3 W for the dose-escalation Q3 W part, until toxicity, disease progression, or any other discontinuation criteria (consent withdrawal, unacceptable adverse events [AEs], poor compliance to study protocol, or patient lost to follow-up). Intra-patient dose-escalation and re-escalation were not allowed for any part.

Tusamitamab ravtansine was administered intravenously at the starting dose of 80 mg/m^2^ in main dose-escalation Q2 W part; additional DLs assessed are mentioned in Table 1. For dose-escalation *bis* part with LD at Cycle 1 followed by 100 mg/m2 Q2 W, the investigation of LD levels with mTPI-2 design was planned for 120 mg/m^2^, 135 mg/m^2^, 150 mg/m^2^, 170 mg/m^2^, and 190 mg/m^2^ (Table 1), followed by treatment with the MTD as 100 mg/m^2^ Q2 W cycle. The starting level of LD was 135 mg/m^2^; doses up to 170 mg/m^2^ were investigated. Two of the three DLT-evaluable patients at the 190 mg/m^2^ DL experienced a DLT in the FIH study. Hence, 190 mg/m^2^ was not evaluated in this study. Dose-escalation was expected to proceed with 150 mg/m^2^ as the starting DL in dose-escalation Q3 W part (Table 1**)**.

**Table 1:** Dose levels

Dose level	Dose of tusamitamab ravtansine (mg/m^2^)		
	Main Dose Escalation Q2 W Part	Dose Escalation Bis Part with Loading Dose followed by 100 mg/m^2^ Q2 W	Dose Escalation Q3 W Part
1	80^a^	–	150^a^
2	100	–	170
L-DL-1	–	120	–
L-DL1	–	135^a^	–
L-DL2	–	150	–
L-DL3	–	170	–
L-DL4	–	190	–

*DL* dose level; *L* loading; *Q2 W* every two weeks; *Q3 W* every three weeks

^a^Starting dose level

### Objectives and endpoints

#### Objectives

The primary study objective was to evaluate the tolerability and safety of tusamitamab ravtansine administered as a single agent according to the investigational medicinal product (IMP)-related DLTs to determine the recommended dose of tusamitamab ravtansine in Japanese patients with advanced malignant solid tumors. The key secondary objectives were to characterize the overall safety profile, PK, and pharmacodynamics (PDy) of tusamitamab ravtansine monotherapy. Preliminary efficacy, as per the Response Evaluation Criteria in Solid Tumors (RECIST) 1.1, and the potential immunogenicity of tusamitamab ravtansine were also evaluated. The tertiary objective was to explore the link between tumor-specific CEACAM5 expression and anti-tumor activity of tusamitamab ravtansine monotherapy.

#### Endpoints

The primary endpoint was the incidence of IMP-related DLTs at C1 and C2 for the main dose-escalation part and dose-escalation *bis* part with LD, and at C1, for the dose-escalation Q3 W part. The key secondary endpoints included the characterization of adverse events (AEs) or other abnormalities, the assessment of PK parameters after single and repeat doses, the assessment of CEACAM5 expression, and the presence of anti-drug antibodies. Efficacy was documented by tumor response, duration of response, and time-to-progression (TTP). Detailed objectives and endpoints are mentioned in Supplementary Table 2.

### Assessments

Safety was assessed by a physical examination, laboratory test abnormalities, and TEAEs as graded by the National Cancer Institute Common Terminology Criteria for Adverse Events (NCI CTCAE) v4.03. AEs were coded to a Preferred Term (PT) and the associated primary System Organ Class (SOC) using the Medical Dictionary for Regulatory Activities (MedDRA 25.1), and summarized by type, frequency, severity, seriousness, and relatedness. Tusamitamab ravtansine concentrations in plasma were determined using a validated immunoassay detecting the ADC bearing at least one DM4 molecule. PK parameters at C1 were calculated by a standard non-compartmental analysis. Tumor response, documented as complete response (CR), partial response (PR), stable disease (SD), or progressive disease (PD), was assessed using RECIST v1.1 if patients had measurable disease at baseline.

### Statistical analysis

All analyses were descriptive unless otherwise specified and performed based on the all-treated population. DLT-evaluable patients had completed two cycles and received at least 80% of the intended dose at each of the first two infusions unless they discontinued the IMP before the completion of C2 due to a DLT. In the dose-escalation Q3 W cohort, all treated population had completed C1 and received at least 80% of the intended dose at the first infusion unless they discontinued the IMP before the end of C1 due to a DLT. Continuous data were summarized using the number of available data, mean, standard deviation, median, minimum, and maximum for each DL. Categorical and ordinal data were summarized using the number and percentage of patients in each DL.

## Results

### Patient disposition and baseline characteristics

**Fig. 2:**
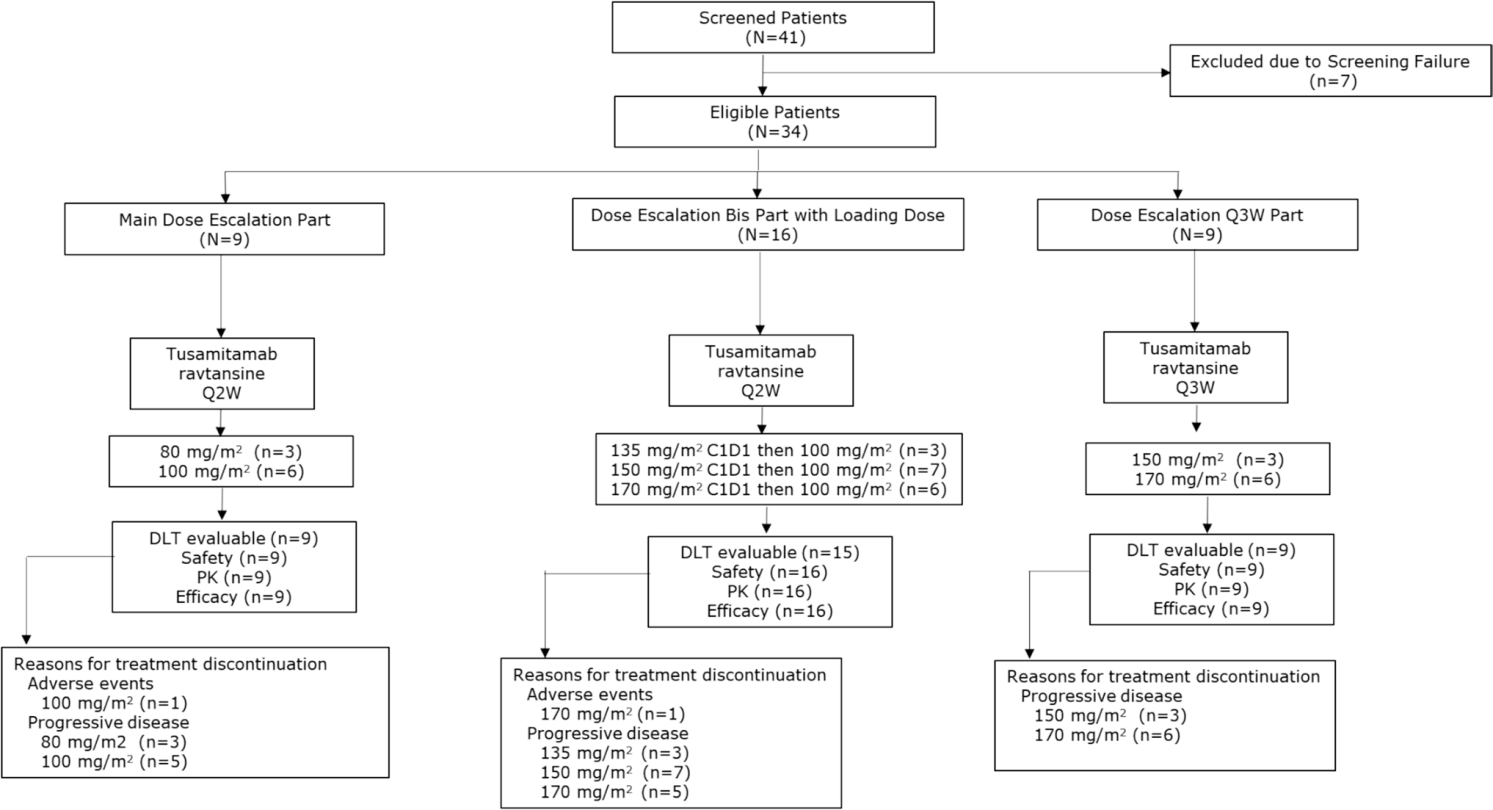
Patient disposition. IMP was continued whenever possible in accordance with the investigator’s judgment and patient consent. *DLT* dose-limiting toxicity; *PK* pharmacokinetics; *Q2 W* every two weeks; *Q3 W* every three weeks

Nine patients were enrolled in the main dose-escalation part, 16 patients in the dose-escalation *bis* part with LD, and nine patients in the dose-escalation Q3 W part. All patients had discontinued treatment in all three parts, with majority of the patients having disease progression (Fig. 2). At the last study contact, no deaths were reported for the main dose-escalation and dose-escalation *bis* parts. In the Q3 W part, one patient had died by the time of the last study contact. The number of patients included in each analysis population is given in Supplementary Table 3.

The median age was 68.0 years and 57.0 years for the main and Q3 W parts and *bis* part, respectively. Most patients in the main Q2 W part were 65 to 75 years of age (77.8%), whereas most patients in the dose-escalation part were < 65 years of age (68.8%). In the Q3 W part, most patients were 65–75 years of age (66.7%) (Table 2). In the main Q2 W part, 88.9% patients had an ECOG score of 0; 68.8% patients in the dose-escalation *bis* part and 77.8% patients in the Q3 W part had an ECOG score of 0).

**Table 2:** Baseline characteristics – All treated population

	(N = 9)		Dose Escalation Bis Part with Loading Dose followed by 100 mg/m^2^ Q2 W (N = 16)			Dose Escalation Q3 W Part (N = 9)	
	80 mg/m^2^ Q2 W (n = 3)	100 mg/m^2^ Q2 W (n = 6)	135 mg/m^2^C1D1, then 100 mg/m^2^ Q2 W (n = 3)	150 mg/m^2^ C1D1, then 100 mg/m^2^ Q2 W (n = 7)	170 mg/m^2^ C1D1, then 100 mg/m^2^ Q2 W (n = 6)	150 mg/m^2^ Q3 W (n = 3)	170 mg/m^2^ Q3 W (n = 6)
Age (years)							
Median	66.0	69.0	50.0	55.0	59.0	57.0	69.0
(min; max)	(41; 70)	(53; 72)	(49; 59)	(48; 74)	(51; 72)	(51; 68)	(57; 74)
Age, n (%)							
< 65	1 (33.3)	1 (16.7)	3 (100)	4 (57.1)	4 (66.7)	2 (66.7)	1 (16.7)
65–75	2 (66.7)	5 (83.3)	0	3 (42.9)	2 (33.3)	1 (33.3)	5 (83.3)
≥ 75	0	0	0	0	0	0	0
Gender, n (%)							
Male	0	4 (66.7)	3 (100)	3 (42.9)	4 (66.7)	1 (33.3)	5 (83.3)
Female	3 (100)	2 (33.3)	0	4 (57.1)	2 (33.3)	2 (66.7)	1 (16.7)
Race, n (%)							
American Indian or Alaska Native	0	0	0	0	0	0	0
Asian	3 (100)	6 (100)	3 (100)	7 (100)	6 (100)	3 (100)	6 (100)
Black/African or African American	0	0	0	0	0	0	0
Native Hawaiian or Other Pacific Islander	0	0	0	0	0	0	0
White/Caucasian	0	0	0	0	0	0	0
Unknown	0	0	0	0	0	0	0
Ethnicity, n (%)							
Hispanic or Latino	0	0	0	0	0	0	0
Not Hispanic or Latino	3 (100)	6 (100)	3 (100)	7 (100)	6 (100)	3 (100)	6 (100)
Unknown	0	0	0	0	0	0	0
ECOG PS							
0	2 (66.7)	6 (100)	3 (100)	3 (42.9)	5 (83.3)	2 (66.7)	5 (83.3)
1	1 (33.3)	0	0	4 (57.1)	1 (16.7)	1 (33.3)	1 (16.7)
Body weight, kg							
Median	53.2	68.4	61.0	53.5	63.3	56.4	64.8
(min; max)	(51.5; 56.1)	(54.8; 90.2)	(60.0; 73.0)	(43.2; 69.2)	(47.0; 68.2)	(40.5; 72.2)	(52.8; 80.9)
BSA (m^2^)							
Median	1.54	1.75	1.76	1.54	1.69	1.54	1.78
(min; max)	(1.5; 1.6)	(1.5; 2.0)	(1.6; 1.8)	(1.4; 1.8)	(1.4; 1.8)	(1.4; 1.9)	(1.4; 1.9)
Prior regimens							
Median	7.0	4.0	5.0	6.0	5.5	4.0	5.0
(min; max)	(5; 11)	(3; 8)	(4; 6)	(4; 9)	(4; 7)	(1; 8)	(3; 7)
Staging at initial diagnosis			0		0		
I	0	0	1 (33.3)	0	1 (16.7)	0	0
II	0	0	0	1 (14.3)	3 (50.0)	1 (33.3)	1 (16.7)
III	1 (33.3)	2 (33.3)	2 (66.7)	3 (42.9)	1 (16.7)	0	1 (16.7)
IV	2 (66.7)	4 (66.7)	0	3 (42.9)	1 (16.7)	2 (66.7)	4 (66.7)
Unknown	0	0		0		0	0
Duration of IMP exposure (weeks)							
Median	14	11	8.86	8.00	8.29	6.43	9.43
(min; max)	(4; 16.1)	(8.1; 16.1)	(8.1; 28.1)	(2; 17.1)	(2; 29)	(6.3; 10.6)	(6; 21.3)
Primary tumor location, n (%)							
Cecum	1 (33.3)	1 (16.7)	0	1 (14.3)	0		
Colon	0	2 (33.3)	0	2 (28.6)	2 (33.3)	1 (33.3)	2 (33.3)
Gastroesophegal junction	0	0	1 (33.3)	0	0		
Pancreas	0	0	0	1 (14.3)	0	1 (33.3)	
Rectum	2 (66.7)	3 (50.0)	2 (66.7)	3 (42.9)	4 (66.7)	1 (33.3)	3 (50.0)
Liver	0	0	0	0	0		0
Extent at study entry, n (%)						2 (66.7)	5 (83.3)
Metastatic*	3 (100)	5 (83.3)	3 (100)	5 (71.4)	6 (100)	1 (33.3)	1 (16.7)
Locally advanced	0	1 (16.7)	0	2 (28.6)	0		

*BSA* body surface area; *C* cycle; *D* day; *ECOG PS* Eastern cooperative oncology group performance status; *IMP* investigational medicinal product; *Q2 W* every two weeks; *Q3 W* every three weeks

^*^Sites included bone, liver, lung, lymph node, rectum, colon, gastroesophageal junction, peritoneum, pleura, cecum

The median prior regimens were 5.0 (range 3–11; main part), 6.0 (range 4–9; *bis* part), and 4.0 (range, 1–8; Q3 W part) (Table 2). The median duration of study treatment was 12 weeks (range 4–16.1; main part), 8.1 weeks (range 2–29; *bis* part), and 6.71 weeks (range 6–21.3; Q3 W part) (Table 2). In the main dose-escalation part, none of the patients had a dose reduction, one patient had a dose interruption, and two patients had a cycle delay of 4 and 7 days. For the dose-escalation *bis* part with LD, one patient had a dose reduction, two patients had a dose interruption, and five patients had a cycle delay. For the Q3 W part, none of the patients had a dose reduction or a dose interruption. Four patients had a cycle delay due to TEAEs.

### Safety and tolerability

#### Main dose-escalation part

Most patients (8/9 [88.9%]) reported a TEAE during the study. Two patients (22.2%) experienced at least one ≥ Grade 3 TEAE, and one patient (11.1%) reported a serious adverse event due to study treatment. One patient (11.1%) with a study-related TEAE discontinued treatment (Table 3). There were no TEAEs leading to death and no DLTs were reported during the observation period.

**Table 3:** Overview of safety profile

n (%)	Main Dose Escalation Part Q2 W (N = 9)		Dose Escalation Bis Part with Loading Dose followed by 100 mg/m^2^ Q2 W (N = 16)			Dose Escalation Q3 W Part (N = 9)	
	80 mg/m^2^ Q2 W (n = 3)	100 mg/m^2^ Q2 W (n = 6)	135 mg/m^2^ C1D1, then 100 mg/m^2^ Q2 W (n = 3)	150 mg/m^2^ C1D1, then 100 mg/m^2^ Q2 W (n = 7)	170 mg/m^2^ C1D1, then 100 mg/m^2^ Q2 W (n = 6)	150 mg/m^2^ Q3 W (n = 3)	170 mg/m^2^ Q3 W (n = 6)
Participants with any TEAE	2 (66.7)	6 (100)	3 (100)	7 (100)	6 (100)	3 (100)	6 (100)
Participants with any related TEAE	1 (33.3)	5 (83.3)	2 (66.7)	7 (100)	5 (83.3)	2 (66.7)	5 (83.3)
Participants with any ≥ Grade 3 TEAE	1 (33.3)	1 (16.7)	2 (66.7)	3 (42.9)	3 (50.0)	2 (66.7)	3 (50.0)
Participants with any related ≥ Grade 3 TEAE	1 (33.3)	0	0	1 (14.3)	1 (16.7)	0	1 (16.7)
Participants with DLT during DLT observation period	0	0	0	1 (14.3)	1 (16.7)	0	0
Participants with any treatment emergent SAE	1 (33.3)	0	1 (33.3)	1 (14.3)	3 (50.0)	2 (66.7)	1 (16.7)
Participants with any related treatment emergent SAE	1 (33.3)	0	0	0	1 (16.7)	0	0
Participants with any TEAE leading to death	0	0	0	0	0	0	1 (16.7)
Participants with any related TEAE leading to death	0	0	0	0	0	0	0
Participants with any TEAE leading to treatment discontinuation	0	1 (16.7)	0	0	2 (33.3)	0	0
Participants with any related TEAE leading to treatment discontinuation	0	1 (16.7)	0	0	1 (16.7)	0	0

*DLT* dose limiting toxicity; *Q2 W* every two weeks; *Q3 W* every three weeks; *SAE* serious adverse event; *TEAE* treatment emergent adverse event

#### Dose-escalation bis part with loading dose

All patients reported TEAEs during the study period. Eight patients (50%) experienced at least one ≥ Grade 3 TEAE, and five patients (31.3%) reported a serious TEAE. One patient (6.3%) had a treatment-related TEAE leading to treatment discontinuation (Table 3). There were no TEAEs leading to death. DLTs were reported in two patients during the DLT observation period at 150 mg/m^2^ and 170 mg/m^2^. One patient at 150 mg/m^2^ LD group reported a Grade 2 keratitis at C2, which was treatment-related and led to a delayed dose. The event was considered resolved. Another patient at 170 mg/m^2^ LD group reported a treatment-related Grade 3 TEAE of increased aspartate aminotransferase (AST) and alanine aminotransferase (ALT) at C1, and a non-serious Grade 3 TEAEs of increased blood alkaline phosphatase and gamma-glutamyl transferase, all of which led to study withdrawal. The event was considered stabilized.

#### Dose-escalation Q3 W part

All patients reported TEAEs during the study period. Five patients (55.6%) experienced at least one ≥ Grade 3 TEAE. Three patients (33.3%) had at least one treatment emergent serious adverse event (TESAE), and one patient (11.1%) experienced a TEAE leading to death due to disease progression (Table 3**)**. No DLTs were reported during the evaluation period. For all parts, AEs by PT and SOC are given in Supplementary Tables 4–6.

#### Adverse events of special interest (AESI)

Three patients (33.3%) in the main dose-escalation part and nine patients (56.3%) in the dose-escalation *bis* part had Grade 1 or 2 treatment-emergent corneal AEs. Four patients (44.4%) in the dose-escalation Q3 W part experienced increased AST (three patients) and pneumonitis (one patient).

### Pharmacokinetics

**Fig. 3:**
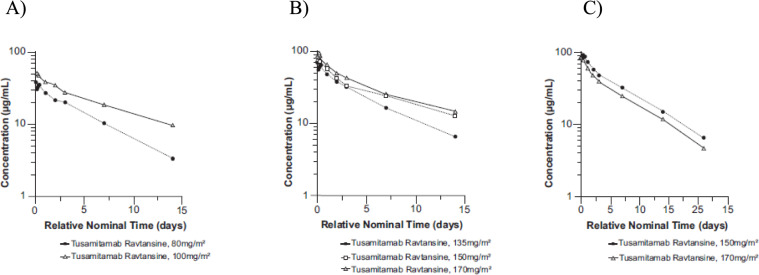
Tusamitamab ravtansine mean PK profile at C1 (semi-log scale). **A** Main dose-escalation part; **B** dose-escalation *bis* part; **C** dose-escalation Q3 W part. *C* cycle; *Q3 W* every three weeks

**Fig. 4:**
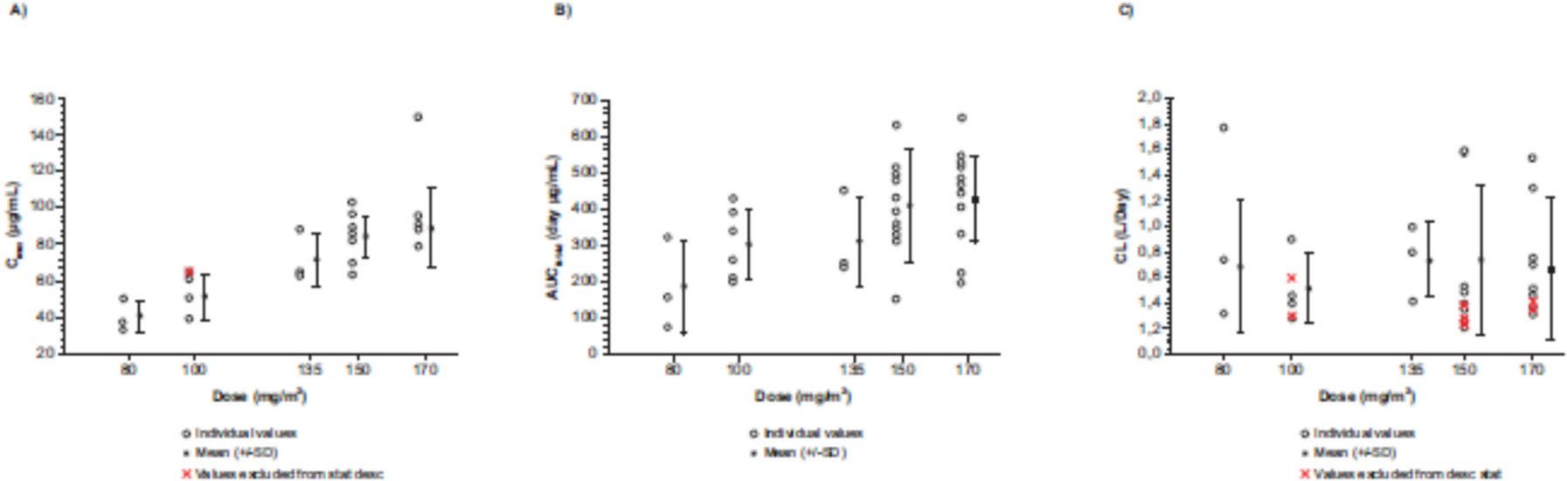
Tusamitamab ravtansine individual and mean (± SD). **A** C_max_, **B** AUC_0–14 d_, and **C** clearance as function of the dose at C1—pool of all parts. *AUC* area under the curve; *C* cycle; *C*_*max*_ maximum concentration; *d* day

Following the first administration, tusamitamab ravtansine was quantifiable in all patients up to D14 (Q2 W) and D21 (Q3 W) for all doses. Mean tusamitamab ravtansine PK profiles during C1 are shown in Fig. 3. Descriptive statistics of tusamitamab ravtansine PK parameters are listed in Table 4. Tusamitamab ravtansine maximum concentration (C_max_) and the area under the curve (AUC) increased in a dose-proportional manner over the dose range of 80–170 mg/m^2^ with an overlap of individual tusamitamab ravtansine exposure across doses. Clearance was constant across doses with an average value of 0.605 L/day (90% confidence interval [CI]: 0.481–0.760 L/day) (Fig. 4).

**Table 4:** Tusamitamab ravtansine PK parameters on Cycle 1 for all three parts

Dose									
	N	C_eoi_ (µg/mL)	C_max_ (µg/mL)	t_max_^a^ (day)	AUC_tau_ (µg·day/mL)	AUC (µg·day/mL)	t_1/2z_ (day)	CL (L/day)	V_ss_ (L)
Main dose escalation part Q2 W									
80 mg/m^2^	3	38.3 ± 10.4	40.1 ± 8.90	0.04	183 ± 127	210 ± 173	3.44 ± 2.30	0.942 ± 0.755	3.18 ± 0.788
		(37.4) [27]	(39.5) [22]	(0.04–0.19)	(154) [69]	(165) [82]	(2.97) [67]	(0.741) [80]	(3.12) [25]
100 mg/m^2^	6	49.8 ± 13.2	51.0 ± 11.9 ^b^	0.13	301 ± 94.4	387 ± 146 ^c^	6.96 ± 1.53	0.513 ± 0.273 ^c^	3.93 ± 0.936 ^c^
		(48.2) [27]	(49.9) [23]	(0.05–0.26)	(289) [31]	(365) [38]	(6.80) [22]	(0.467) [53]	(3.85) [24]
Dose escalation bis part Q2 W									
135 mg/m^2^	3	68.8 ± 12.3	71.6 ± 14.5	0.12	308 ± 120	372 ± 198	4.87 ± 2.18	0.736 ± 0.294	4.19 ± 0.441
		(68) [18]	(70.7) [20]	(0.05–0.13)	(294) [39]	(341) [53]	(4.56) [45]	(0.690) [40]	(4.17) [11]
150 mg/m^2^	7	73.9 ± 11.5	80.2 ± 9.87	0.13	363 ± 154	359 ± 250 ^d^	6.76 ± 4.18	1.02 ± 0.651 ^d^	3.18 ± 0.674 ^d^
		(73.1) [16]	(79.6) [12]	(0.04–0.32)	(325) [42]	(289) [70]	(4.99) [62]	(0.846) [64]	(3.13) [21]
170 mg/m^2^	6	74.7 ± 12.1	97.1 ± 26.7	0.17	444 ± 125	535 ± 240 ^c^	6.90 ± 2.60	0.712 ± 0.551^c^	4.32 ± 0.641^c^
		(73.9) [16]	(94.6) [28]	(0.05–0.21)	(423) [28]	(472) [45]	(6.16) [38]	(0.595) [77]	(4.29) [15]
Dose escalation Q3 W part									
150 mg/m^2^	3	87.9 ± 13.1	93.8 ± 10.9	0.06	593 ± 142	688 ± 182	7.56 ± 1.57	0.363 ± 0.117	3.56 ± 1.67
		(87.3) [15]	(93.4) [12]	(0.05–0.29)	(582) [24]	(673) [26]	(7.44) [21]	(0.351) [32]	(3.32) [47]
170 mg/m^2^	6	77.6 ± 15.8	80.5 ± 13.0	0.14	475 ± 162	549 ± 236	6.54 ± 2.41	0.647 ± 0.359	4.54 ± 0.904
		(76.2) [20]	(79.6) [16]	(0.04–0.29)	(448) [34]	(503) [43]	(6.18) [37]	(0.576) [55]	(4.46) [20]

^a^Median (min; max)

^b^N = 5

^c^N = 4

^d^N = 4

*AUC*_*tau*_ AUC_0-14 d_ (Q2 W) or AUC_0-21 d_ (Q3 W)AUC, area under curve extrapolated to infinity; *C*_*eoi*_ concentration at the end of the infusion; *C*_*max*_ maximum plasma concentration; *PK* pharmacokinetics; *Q2 W* every two weeks; *Q3 W* every three weeks; *SD* standard deviation; *t*_*1/2*_ terminal half-life; *t*_*max*_ maximum drug concentration time

#### Comparison of PK in multiregional first-in-human and Japanese studies

**Fig. 5:**
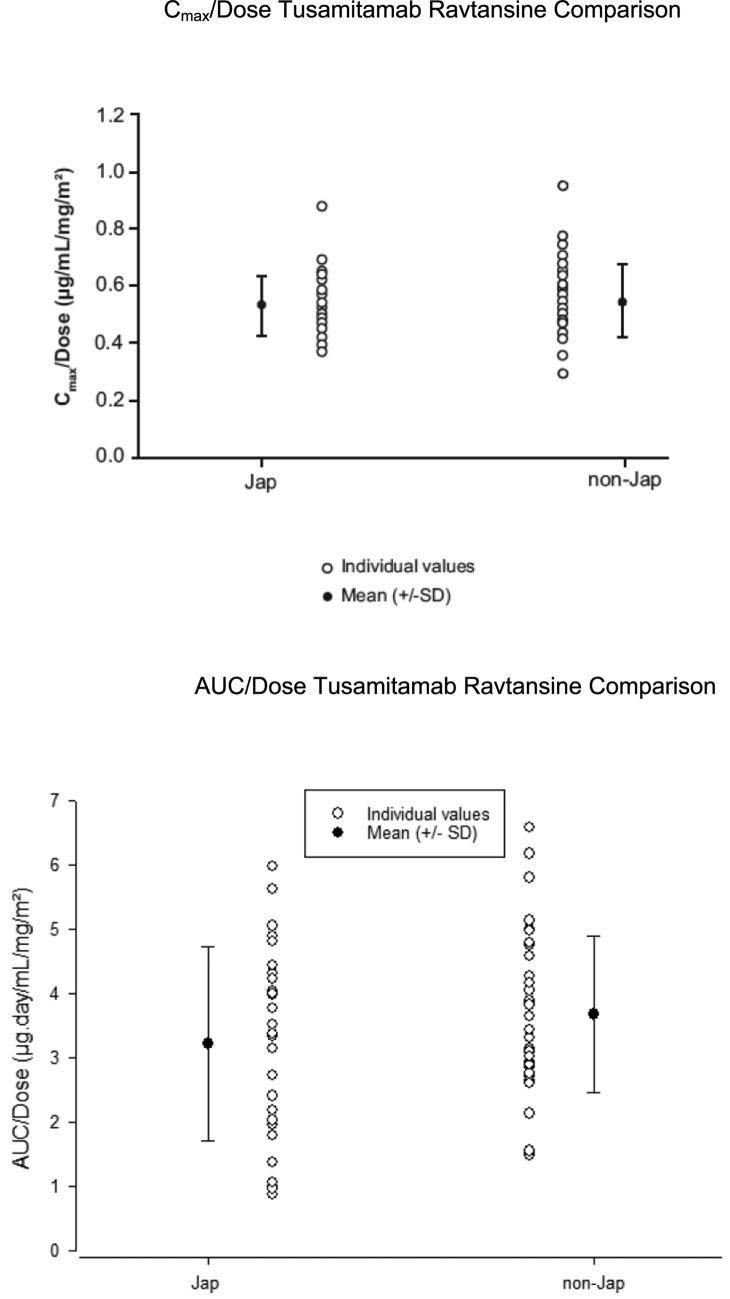
Comparison of tusamitamab ravtansine PK parameters (C_max_ and AUC) normalized by dose between Japanese and non-Japanese patients at Cycle 1. *AUC* area under the curve; *C* cycle; *C*_*max*_ maximum concentration; *Jap* Japanese; *SD* standard deviation

Due to the small number of patients at each DL for all parts in the multiregional FIH and Japanese studies, the exposure PK parameters were normalized by DL for comparison; no difference was observed in the exposure parameters (Fig. 5).

### Anti-tumor activity

#### Main dose-escalation part

**Fig. 6:**
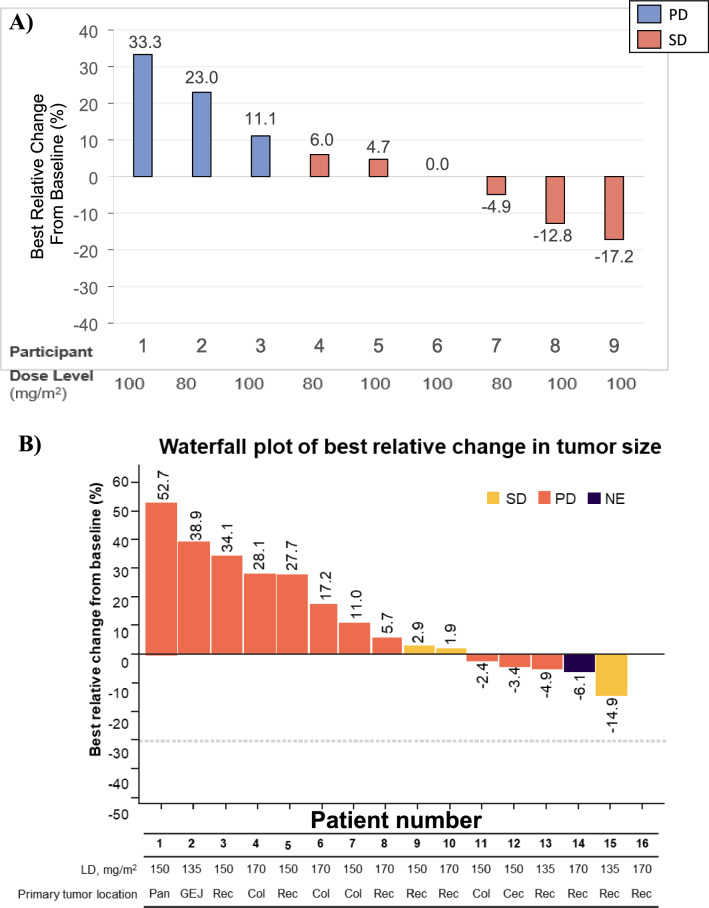

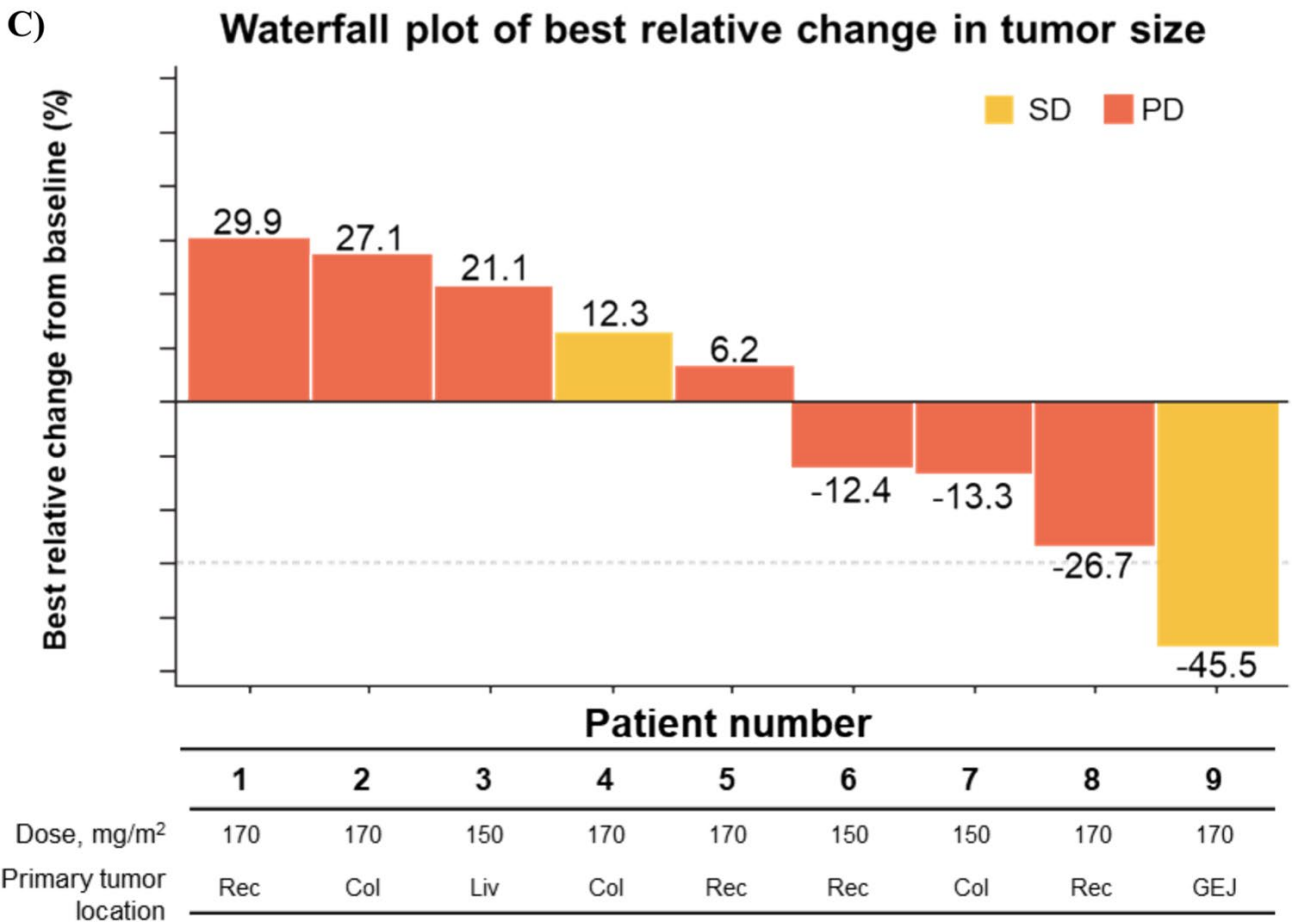
Waterfall plot of best relative change in tumor size. **A** Main dose-escalation part. **B** Dose-escalation *bis* part. **C** Dose-escalation Q3 W part. *Col* colon; *GEJ* gastroesophageal junction adenocarcinoma; *LD* loading dose; *NE* not evaluable; *Pan* pancreatic; *PD* progressive disease; *Q3 W* every three weeks; *Rec* rectum; *SD* stable disease

The best overall response (BOR) was assessed in three patients at 80 mg/m^2^ and six patients at 100 mg/m^2^; no responders were observed at either DL. Two patients (66.7%) at 80 mg/m^2^ and four patients (66.7%) at 100 mg/m^2^ had SD and remaining had PD (Table 5; Fig. 6a). The median TTP was 3.06 months (95% CI: 1.906–3.713).

**Table 5:** Summary of best overall response – All treated population

n (%)	(N = 9)		Dose Escalation Bis Part with Loading Dose followed by 100 mg/m^2^ Q2 W (N = 16)			Dose Escalation Q3 W Part (N = 9)	
	80 mg/m^2^ Q2 W (n = 3)	100 mg/m^2^ Q2 W (n = 6)	135 mg/m^2^ C1D1, then 100 mg/m^2^ Q2 W (n = 3)	150 mg/m^2^ C1D1, then 100 mg/m^2^ Q2 W (n = 7)	170 mg/m^2^ C1D1, then 100 mg/m^2^ Q2 W (n = 6)	150 mg/m^2^ Q3 W (n = 3)	170 mg/m^2^ Q3 W (n = 6)
Best overall response, n (%)							
Complete response	0	0	0	0	0	0	0
Partial response	0	0	0	0	0	0	0
Stable disease	2 (66.7)	4 (66.7)	1 (33.3)	1 (14.3)	1 (16.7)	0	2 (33.3)
Progressive disease	1 (33.3)	2 (33.3)	2 (66.7)	6 (85.7)	4 (66.7)	3 (100)	4 (66.7)
Not evaluable	0	0	0	0	1 (16.7)	0	0
Responders (CR or PR)	0	0	0	0	0	0	0
95% CI^a^	(0.0; 70.7)	(0.0; 45.9)	(0.0; 70.7)	(0.0; 40.9)	(0.0; 45.9)	(0.0; 70.7)	(0.0; 45.9)

^a^estimated by Clopper-Pearson exact method

*C* cycle; *CI* confidence interval; *CR* complete response; *D* day; *PR* partial response; *Q2 W* every two weeks; *Q3 W* every three weeks

#### Dose-escalation bis part with loading-dose

There were no responders in this part at any DL. One patient at each DL had the BOR of SD and remaining had PD (Fig. 6b). One patient (16.7%) at 170 mg/m^2^ was not evaluable (Table 5). The median TTP was 1.77 months (95% CI: 1.676–2.136).

#### Dose-escalation Q3 W part

The BOR was assessed in three patients treated at 150 mg/m^2^ and six patients treated at 170 mg/m^2^; no responders were reported at any DL. Two patients (33.3%) at 170 mg/m^2^ had the BOR of SD, and all other patients had PD (Table 5; Fig. 6c). The median TTP was 2.00 months (95% CI: 1.413–2.825).

## Discussion

Tusamitamab ravtansine monotherapy was generally well tolerated in all three parts investigated in this study, and no new safety concerns were identified relative to our previous study [10, 11]. DLTs were not observed during the observation period, except for two events reported in the dose-escalation *bis* part: one in the 150 mg/m^2^ LD group (resolved) and one in the 170 mg/m^2^ LD group (stabilized). Considering all the three parts of this study, tusamitamab ravtansine exposure (C_max_, AUC_0–14 d_, and AUC) increased in a dose-proportional manner from 80 to 170 mg/m^2^, and the overall mean clearance of tusamitamab ravtansine was approximately 0.6 L/day. There were no patients with CR or PR according to the RECIST v1.1; the BOR of SD was observed in all three parts.

The most common TEAEs were gastrointestinal disorders, corneal events, and metabolic disorders, similar to the FIH study [10, 11]. Blurred vision and keratitis resulted in treatment discontinuation and dose modification, respectively. Ocular adverse events are commonly reported with the administration of ADCs, especially with ADCs employing maytansinoids [12]. In the FIH study, Grade 2 or 3 keratopathy and keratitis were reported [10, 11]. Further, Eaton et al. analyzed 22 studies reporting ocular or vision-impairing AEs following treatment with different ADCs, commonly leading to dose modification [12]. Keratitis (8/22), dry eye (7/22), corneal microcysts (5/22), corneal deposits/inclusions (4/22), conjunctivitis/keratoconjunctivitis (3/22), and unspecified keratopathy (2/22) were the most frequently reported AEs in these studies. The mechanism(s) leading to corneal events and the role of structural and pharmacological properties of ADCs warrant further investigation [12].

An increase in AST and ALT (Grade ≥ 3) was reported in three patients in the dose-escalation *bis* part and six patients in the Q3 W part. Similarly, a Grade 3 transaminase elevation was observed in the FIH study [10, 11]. Abnormal liver enzyme levels have previously been reported as an ADC-related AE. In the Phase 3 EMILIA study, Diéras et al. also observed elevated AST levels in 22 of 490 (5%) patients with HER2-positive metastatic breast cancer treated with the ADC trastuzumab emtansine [13]. In their meta-analysis, Zhu et al. highlighted the need for special attention to liver enzyme levels following treatment with ADCs via the administration of appropriate drugs that lower liver enzyme levels [14].

The exposure parameters for tusamitamab ravtansine overlapped between Japanese and non-Japanese patients, and no difference in PK was evidenced between the two populations. Further, it was not clear as to why the objective response was not observed among the patient population; a high number of prior regimens resulting in refractory disease could be one of the contributing factors. In the FIH study as well, the objective response was observed in only three patients from the Q2 W fixed-dosing regimen, while no response was reported in the Q2 W-LD or Q3 W cohort [10, 11]. Hence, anti-tumor activity may be observed with tusamitamab ravtansine in select dosing regimen.

In conclusion, this Phase 1 dose-escalation study demonstrated that tusamitamab ravtansine was well tolerated at a dose of 80 to 170 mg/m^2^ in Japanese patients with metastatic or locally advanced solid tumors. In further investigation of tusamitamab ravtansine in a global, randomized, open-label Phase 3 trial with previously treated patients with advanced non-squamous NSCLC (CARMEN-LC03), overall survival demonstrated a trend favoring tusamitamab ravtansine; however, the trial did not meet its dual primary endpoint of progression-free survival [15]. While tusamitamab ravtansine program was terminated in December 2023, our data provide initial clinical evidence of the relevance of CEACAM5 targeted therapies for patients with advanced NSCLC [16].

## Data Availability

Qualified researchers can request access to patient-level data and related study documents, including the clinical study report, study protocol with any amendments, blank case report forms, statistical analysis plan, and dataset specifications. Patient-level data will be anonymized, and study documents will be redacted to protect the privacy of trial patients. Further details on Sanofi’s data-sharing criteria, eligible studies, and process for requesting access are available online.
